# EndoS_2_ is a unique and conserved enzyme of serotype M49 group A *Streptococcus* that hydrolyses N-linked glycans on IgG and α_1_-acid glycoprotein

**DOI:** 10.1042/BJ20130126

**Published:** 2013-09-13

**Authors:** Jonathan Sjögren, Weston B. Struwe, Eoin F. J. Cosgrave, Pauline M. Rudd, Martin Stervander, Maria Allhorn, Andrew Hollands, Victor Nizet, Mattias Collin

**Affiliations:** *Department of Clinical Sciences, Division of Infection Medicine, Lund University, Biomedical Center B14, SE-22184 Lund, Sweden; †The National Institute for Bioprocess Research and Training, NIBRT, University College Dublin, Fosters Avenue, Mount Merrion, Blackrock, Co. Dublin, Ireland; ‡Molecular Ecology and Evolution Laboratory, Department of Biology, Lund University, Ecology Building, SE-223 62 Lund, Sweden; §School of Biological Sciences, University of Wollongong, Wollongong, NSW 2522, Australia; ∥Department of Pediatrics, University of California San Diego, 9500 Gilman Drive, Mail Code 0687, La Jolla, CA 92093, U.S.A.; ¶Skaggs School of Pharmacy and Pharmaceutical Sciences, University of California San Diego, 9500 Gilman Drive, Mail Code 0687, La Jolla, CA 92093, U.S.A.

**Keywords:** α_1_-acid glycoprotein, endo-β-N-acetylglucosaminidase, host–pathogen interaction, IgG glycosylation, *Streptococcus pyogenes*, 2-AB, 2-aminobenzamide, ABS, *Arthrobacter ureafaciens* sialidase, AGP, α_1_-acid glycoprotein, AMF, almond meal α-fucosidase, BEH, bridged ethane–silicon hybrid, BKF, bovine kidney α-fucosidase, BTG, bovine testes β-galactosidase, CM, C-medium, CcpA, catabolite control protein A, FcγR, Fcγ receptor, FLD, fluorescence detection, GAS, group A *Streptococcus*, GH18, family 18 of glycoside hydrolases, HILIC, hydrophilic interaction liquid chromatography, HRP, horseradish peroxidase, LCA, *Lens culinaris* agglutinin, 4MU-GlcNAc, 4-methylumbelliferyl *N*-acetyl-β-D-glucosaminide, MWCO, molecular-mass cut-off, NAN1, neuraminidase/sialidase 1, PNGase F, peptide N-glycosidase F, r, recombinant, UHPLC, ultra-HPLC

## Abstract

Many bacteria have evolved ways to interact with glycosylation functions of the immune system of their hosts. *Streptococcus pyogenes* [GAS (group A *Streptococcus*)] secretes the enzyme EndoS that cleaves glycans on human IgG and impairs the effector functions of the antibody. The *ndoS* gene, encoding EndoS, has, until now, been thought to be conserved throughout the serotypes. However, in the present study, we identify EndoS_2_, an endoglycosidase in serotype M49 GAS strains. We characterized EndoS_2_ and the corresponding *ndoS2* gene using sequencing, bioinformatics, phylogenetic analysis, recombinant expression and LC–MS analysis of glycosidic activity. This revealed that EndoS_2_ is present exclusively, and highly conserved, in serotype M49 of GAS and is only 37% identical with EndoS. EndoS_2_ showed endo-β-N-acetylglucosaminidase activity on all N-linked glycans of IgG and on biantennary and sialylated glycans of AGP (α_1_-acid glycoprotein). The enzyme was found to act only on native IgG and AGP and to be specific for free biantennary glycans with or without terminal sialylation. GAS M49 expression of EndoS_2_ was monitored in relation to carbohydrates present in the culture medium and was linked to the presence of sucrose. We conclude that EndoS_2_ is a unique endoglycosidase in serotype M49 and differs from EndoS of other GAS strains by targeting both IgG and AGP. EndoS_2_ expands the repertoire of GAS effectors that modify key glycosylated molecules of host defence.

## Abstract 2

In this study, the endoglycosidase EndoS_2_ was characterized. The enzyme was found to be unique and conserved in serotype M49 of group A *Streptococcus* and to specifically cleave N-linked glycans on IgG and AGP.

## INTRODUCTION

Glycosylation is a common post-translational modification, and almost all key molecules in the immune system are glycosylated [1]. IgG is the most abundant antibody in serum with the capacity to bind and neutralize antigens, facilitate antibody-dependent cytotoxicity, opsonize antigens and initiate phagocytosis. IgG is composed of two light and two heavy chains, of which the latter are glycosylated with complex N-linked glycans at Asn^297^. The presence and structure of this glycan is of major importance for the interaction of the antibody with FcγRs (Fcγ receptors) and for the subsequent effector functions elicited by the antibody [2–4]. The glycan is present in a pocket of the two heavy chains of the IgG molecule, where it has been shown to be flexible and dynamic allowing it to influence the glycan–protein interaction with FcγR [5]. IgA, IgD, IgE and IgM each carry several occupied N- and O-linked glycosylation sites, and the study of the glycan's impact on the effector functions of these immunoglobulins has only begun [6].

*Streptococcus pyogenes* [GAS (group A *Streptococcus*)] is a leading Gram-positive bacterial pathogen exhibiting a wide array of immune evasion mechanisms, including interference with host glycosylation [7]. Every year, this bacterium causes over 500000 deaths due to severe infections and post-infectious immunological disorders: invasive infections, rheumatic fever, glomerulonephritis and hundreds of millions of cases of milder and self-limiting infections, such as pharyngitis and impetigo [8]. GAS is subdivided into serotypes on the basis of the antigenic M-protein on the bacterial surface and there are currently over 100 serotypes described [9].

An endoglycosidase from *S. pyogenes*, EndoS, was discovered in serotype M1 of GAS and found to hydrolyse the N-linked glycan on the heavy chain of native human IgG and in this way modulate the binding of IgG to FcγR [10–12]. EndoS (EC 3.2.1.96) belongs to GH18 (family 18 of glycoside hydrolases) and has endo-β-N-acetylglucosaminidase activity (CAZy, 2012; http://www.cazy.org). Enzymes in the family GH18 hydrolyses β-1,4-linked GlcNAc and this group of enzymes contains both chitinases (EC 3.2.1.14) hydrolysing the carbohydrate chitin and endo-β-N-acetylglucosaminidases (EC 3.2.1.96) with described endoglycosidase activity on the chitobiose core of N-linked complex glycans (CAZy, 2012).

EndoS is expressed in late stationary phase during streptococcal growth and the catalytically active glutamate residue (Glu^235^) and several tryptophan residues are required for enzymatic activity [13]. Different from other described bacterial endoglycosidases, EndoS hydrolyses the N-linked glycan only on native and not denatured IgG [14]. Complement activation by the classical pathway was reduced when antibodies were treated with EndoS [11]. In human blood, the recombinant enzyme has been shown to deglycosylate IgG, and, in an opsonophagocytic killing assay, recombinant EndoS was shown to increase bacterial survival [11]. The contribution of EndoS to GAS virulence has been studied in a mouse model of invasive infection, and, although of minor importance in the wild-type M1 bacteria, it increased virulence of other GAS strains when heterologously expressed [15]. As a strategy to treat autoimmune diseases, EndoS has shown promise as a biotherapeutic in a number of animal models of autoimmunity [16–21]. For the biotechnology industry, the enzyme has applications both as a tool in the analysis of monoclonal antibodies (Genovis AB) and potentially for chemoenzymatic glycoengineering [22,23].

In the genome of GAS strain NZ131 of serotype M49, we have identified the gene *ndoS2* encoding the enzyme EndoS_2_ [24]. *ndoS2* holds 53% identity with *ndoS* and the proteins EndoS_2_ and EndoS are 37% identical. The GAS strain NZ131 is a clinical isolate from a case of acute post-streptococcal glomerulonephritis in New Zealand [24]. Serotype M49 belongs to a serotype grouping of GAS associated with skin infections and glomerulonephritis, group II (M2, M42, M49, M56, M57 and M60), rather than throat infections and rheumatic fever (M1, M4, M12 and M25) that define group I [24,25].

In the present study, we characterize EndoS_2_ using bioinformatics, recombinant expression and LC–MS analysis to study the glycosidic activity.

## MATERIALS AND METHODS

### Bacterial strains and growth

The genome of *S. pyogenes* GAS strain NZ131 of serotype M49 has been sequenced and this strain was therefore selected as the reference strain in the present study [24,25]. GAS was propagated on blood agar, *Escherichia coli* strains Top10 (Invitrogen) and BL21 pLysS (Invitrogen) were propagated on lysogeny broth agar and used for cloning and recombinant expression. All strains used are summarized in Supplementary Table S1 (http://www.biochemj.org/bj/455/bj4550107add.htm). For selection in *E. coli* Top10 cells, carbenicillin was used at 100 μg·ml^−1^ and, for *E. coli* BL21 pLysS, 100 μg·ml^−1^ carbenicillin and 34 μg·ml^−1^ chloramphenicol were used. Overnight cultures of *E. coli* were carried out in lysogeny broth at 37°C with aeration. Genomic DNA preparation of GAS strain NZ131 was performed using Puregene DNA Purification Kit (Qiagen). Transformation was carried out using heat-shock at 42°C for 30 s. Plasmid preparations from *E. coli* were performed using Plasmid Miniprep Kit I (Omega Bio-Tek). All primers used are listed in Supplementary Table S2 (http://www.biochemj.org/bj/455/bj4550107add.htm). Expression of EndoS_2_ was studied using growth of NZ131 in 50% CM (C-medium) [0.5% Proteose Peptone, 1.5% (w/v) yeast extract, 10 mM K_2_PO_4_, 0.4 mM MgSO_4_ and 17 mM NaCl (pH 7.5)].

### Sequencing of *ndoS2*

Five GAS serotype M49 strains were selected for sequencing of the *ndoS2* gene; 3487-05, AP49, ACN49, AW1 and AW2. Sequencing was carried out using primers ndoS2-out-R, seq38-R, seq42-R, seq54-R, seq15-F, seq17-F, seq24-F and seq28-F and the Lightrun sequencing service of GATC Biotech (Konstanz, Germany). All primers used for sequencing are summarized in Supplementary Table S2. The sequences have been deposited in GenBank® with accession numbers as follows: KC155346 (strain 3487-05), KC155348 (strain AP49), KC155347 (strain ACN49), KC155349 (strain AW1), KC155350 (strain AW2) (Supplementary Table S2).

### Recombinant expression of EndoS_2_

Recombinant expression of EndoS_2_ in *E.* c*oli* was established by PCR amplification of the *ndoS2* gene from GAS NZ131 with the primers ndoS2-F-BamHI, 5′-CTGTAAGGATCCAGGAGAAGACTG-3′, and ndoS2-R-XhoI, 5′-GAAACCTCGAGTCTTTGTAATCGTAGGACTT-3′. The *ndoS2* fragment was digested with restriction enzymes BamHI and XhoI (restriction sequences are underlined) and ligated into the expression vector pGEX-5X-3 (GE Healthcare) using DNA ligase T4 (Thermo Fisher Scientific) creating the plasmid pGEX-ndoS2. The expression vector was transformed into *E. coli* Top10 chemically competent cells and screened with PCR using primers ndoS2-F-BamHI and ndoS2-R-XhoI. Positive clones were isolated and the pGEX-ndoS2 plasmid was purified and transformed into the *E. coli* expression strain BL21 pLysS. One recombinant clone was grown overnight at 37°C with antibiotics, diluted 1:20 in lysogeny broth medium with antibiotics and grown for 3 h to mid-exponential phase. The expression of the protein GST–EndoS_2_ was induced with 0.1 mM IPTG for 3 h. The cells were harvested and lysed with BugBuster Protein Extraction Reagent (Novagen/Merck). Recombinant GST–EndoS_2_ was purified on a column with glutathione–Sepharose 4B (GE Healthcare) and eluted with reduced glutathione. The GST tag was cleaved off using Factor Xa (New England BioLabs). Site-directed mutagenesis was performed on pGEX-ndoS2 using a QuikChange® II Site-Directed Mutagenesis Kit (Agilent) with primers ndoS2(E-L)-F and ndoS2(E-L)-R, to exchange the glutamate residue (E) in the active site to leucine (L). Recombinant EndoS_2_(E186L) was cloned and expressed in a similar way to EndoS_2_.

### Phylogenetic analysis

We searched non-redundant protein databases at NCBI with the BLASTP algorithm, submitting the EndoS_2_ sequence of GAS strain ACN49 (M49). We retrieved similar protein sequences, setting a cut-off of the expect value at <10^−10^. All but two sequences belonged to GH18, except for two sequences of other hypothetical proteins. All sequences were included in phylogenetic analyses of the proteins, and the non-GH18 proteins served as outgroup. A total of 101 protein sequences were aligned in Geneious version 6.0.3 (Biomatters Ltd, available from http://www.geneious.com/) using the ClustalW algorithm (Supplementary Table S3 at http://www.biochemj.org/bj/455/bj4550107add.htm). From the alignment of 1817 amino acids, a region of generally high coverage comprising 1099 amino acids was extracted and analysed in BEAST version 1.7.4 [7,26]. We used the Blosum62 substitution model [8,27] with no site heterogeneity specification, set a strict molecular clock, selected the Yule process [9,28] for tree prior and ran Bayesian MCMC sampling every 1000 generations for 10 million generations. The output was examined with Tracer version 1.5 (A. Rambaut and A.J. Drummond, available from http://beast.bio.ed.ac.uk/Tracer) in order to ensure that likelihood scores were stationary and that effective sample sizes were adequate (>500), setting burnin to 25%. A maximum clade credibility tree was calculated with TreeAnnotator version 1.7.4 [1,26]. 16S rRNA (RNA or cDNA) sequences were retrieved from the Ribosomal Project Database (http://rdp.cme.msu.edu) and GenBank® for taxonomic analyses of the corresponding taxa/strains in the EndoS dataset (Supplementary Table S3). In case a specific strain was lacking for taxa more distantly related in the EndoS protein tree dataset, another strain was chosen if available. This resulted in a total of 51 representative sequences, which were aligned according to the procedures described for the EndoS protein dataset above. From the resulting alignment of 2172 nt, a high coverage region comprising 1576 positions was extracted and analysed in BEAST version 1.7.4 [2–4,26] with similar parameter settings except for the substitution model [HKY with rate variation across sites following a discrete gamma distribution (G) with four rate categories]. Verification and tree calculation followed the procedures above, and the tree was rooted using the fungal sequences, which were a part of the dataset, as outgroup.

### EndoS_2_ expression analysis

Overnight cultures of NZ131 in CM were diluted 1:50 and grown for 16 h at 37°C with 5% CO_2_ and the cysteine protease inhibitor E-64 at 20 μM. Glucose, galactose, sucrose, acetylglucosamine and mannose were added at 0.01% concentration when indicated. Supernatants were concentrated using precipitation with 0.3 mM TCA (trichloroacetic acid). The samples and 0.5 μg of recombinant EndoS_2_ were resuspended in SDS/PAGE loading buffer and loaded on to a 10% Bis-Tris gel. The electrophoresis was performed at 180 V for approximately 60 min and stained with PageBlue Protein Staining Solution (Fermentas). Blotting on to a PVDF membrane was performed according to the manufacturer's instructions using Trans-Blot Turbo (Bio-Rad Laboratories) equipment. The membrane was blocked in 5% (w/v) dried skimmed milk powder (Difco) and incubated with 10 μl of rabbit polyclonal anti-EndoS_2_ for 1 h at 37°C with rotation. Washing was consistently carried out in PBST (PBS with 0.05% Tween 20) three times for 10 min. The membrane was washed and incubated with 2.5 μl of HRP (horseradish peroxidase)-conjugated goat anti-(rabbit IgG) (H+L) (Bio-Rad Laboratories), washed and developed using Supersignal West Pico Chemiluminescent Substrate (Thermo Fisher Scientific). Antiserum against EndoS_2_ was obtained using 1 mg of recombinant EndoS_2_ to immunize rabbits following standard protocols by Davids Biotechnologie, Regensburg, Germany.

### N-glycan hydrolysis assay

A sample of 1 μg of recombinant EndoS_2_, EndoS_2_(E186L) or EndoS or 500 units of PNGase F (peptide N-glycosidase F) was incubated with 3 μg of IgG or 5 μg of AGP (α_1_-acid glycoprotein) (Sigma–Aldrich) in PBS at 37°C for 2 h. For PNGase F, the substrate was denatured according to the manufacturer's instructions (New England BioLabs). Human IgG subclasses IgG_1–4_ (Calbiochem/Merck) were incubated with recombinant EndoS_2_, EndoS_2_(E186L) or PBS under the reaction conditions described above. All reactions were separated on a 10% Bis-Tris gel as described above. Lectin blotting was performed on a PVDF membrane (Millipore). The membrane was incubated in lectin blot buffer (10 mM Hepes, 0.15 M NaCl, 0.1% Tween 20, 0.01 mM MnCl_2_ and 0.1 mM CaCl_2_) for 1 h and incubated with 5 μg of biotinylated LCA (*Lens culinaris* agglutinin) (Vector Laboratories) in the same buffer. The membrane was washed three times for 10 min in lectin blot buffer and subsequently 2.5 μg of HRP coupled to streptavidin (Vector Laboratories) was added for 1 h. The membrane was developed as described above. The 16 h bacterial supernatants were concentrated using 10 kDa MWCO (molecular-mass cut-off) spin columns (Pall) and a functional assay on the activity of the supernatants on 3 μg of human serum IgG was performed. The reaction mixture was incubated at 37°C overnight and analysed by SDS/PAGE (10% gel) and a subsequent LCA lectin blot as described above.

### Chitinase assay

4MU-GlcNAc (4-methylumbelliferyl *N*-acetyl-β-D-glucosaminide) (Sigma–Aldrich) was incubated at 0.2 mM with 0.3 m-unit of chitinase from *Streptomyces griseus* (Sigma–Aldrich) or 2 μg of rEndoS_2_ (where r denotes recombinant) or 2 μg of rEndoS or PBS in 100 μl of PBS. The reactions were incubated at 37°C for 1 h. Then, 100 μl of 0.1 M glycine (pH 10) was added to stop the reaction. Absorbance at 355/445 nm was measured in a black 96-well plate using a spectrophotometer. The experiments were carried out using five replicates and results are shown as means±S.D. The response in absorbance was analysed statistically by an unpaired Student's *t* test, where differences were considered significant if *P*<0.05. ****P<0.001.

### Glycoprotein denaturing

A 4 μg amount of IgG or AGP was incubated in 10 μl of PBS at 37°C, 40°C, 50°C, 60°C, 70°C or 80°C for 30 min. After the incubation, the samples were kept at 37°C. Then, 2 μg of rEndoS_2_ was added to each reaction mixture and incubated further at 37°C for 2 h. The samples were analysed on a SDS/PAGE gel and for IgG with LCA lectin blotting as described above. For analysis of EndoS_2_ specificity, 4 μg of α_2_-macroglobulin, ovalbumin, human lactoferrin, RNase B and fetuin (all Sigma–Aldrich) were incubated with 2 μg of EndoS_2_ at 37°C overnight and subsequently analysed on SDS/PAGE gel as described.

### LC–FLD (fluorescence detection)–MS

Online coupled LC–MS with FLD was performed using a Waters Xevo G2 QTof with Acquity UPLC and BEH (bridged ethane–silicon hybrid) glycan column (1.0 mm×150 mm, 1.7 μm particle size). MS data was acquired in negative mode with the following conditions: 2500 V capillary voltage, 50 V cone voltage, 280°C desolvation temperature, 600 l·h^−1^ desolvation gas and 100°C source temperature. The analyser was set to sensitivity mode. The fluorescence data rate was 1 point·s^−1^ and a PMT gain of 10 with excitation and emission wavelengths set at 320 nm and 420 nm respectively. Samples were in 80% acetonitrile with an injection volume of 10 μl. The flow rate was 0.150 μl·min^−1^. Solvent A was 50 mM ammonium formate (pH 4.4) and solvent B was acetonitrile. A 40 min linear gradient was used and was as follows: 28–43% solvent A for 31 min, 70% solvent A for 4 min and 28% solvent A for 4 min.

### Exoglycosidase digestion arrays

Analysis of glycan sequence, composition and linkage specificities was facilitated by the use of exoglycosidase digestion arrays. All digestion reactions were performed with enzymes from Prozyme. Fluorescently labelled glycans were digested in 50 mM sodium acetate (pH 5.5) at 37°C overnight using a panel of enzymes with each digestion reaction brought to a final volume of 10 μl using double-distilled water. Digested glycans were then separated from the enzyme mixtures using 10 kDa MWCO centrifugal filters. Digested 2-AB (2-aminobenzamide)-labelled glycans were then prepared for separation on UHPLC (ultra-HPLC) with fluorescence detection using a BEH glycan column as described previously [5,29]. Specific non-reducing end monosaccharides were removed as follows: terminal sialic acid in all linkages was removed with 1 m-unit·μl^−1^ ABS (*Arthrobacter ureafaciens* sialidase); terminal galactose monosaccharides were removed using 0.5 m-unit·μl^−1^ BTG (bovine testes β-galactosidase), which releases both β(1,3)- and β(1,4)-linked galactose; terminal GlcNAc monosaccharides were released with 40 m-unit·μl^−1^ GUH (*Streptococcus pneumoniae* hexosaminidase), capable of cleaving β-linked GlcNAc moieties; core α(1,6)-fucose was selectively removed using 1 m-unit·μl^−1^ BKF (bovine kidney α-fucosidase), (2,3)-linked sialic acid was removed using 10 m-unit·μl^−1^ recombinant *Streptococcus pneumoniae* NAN1 (neuraminidase/sialidase 1), and AMF (almond meal α-fucosidase) at 6 m-unit·μl^−1^ was used to release (1,3)- and (1,4)-linked non-reducing terminal fucose residues.

### Activity of EndoS_2_ on free N-glycans

N-glycans present on 80 μg of bovine fetuin (Sigma–Aldrich) were released using 2500 units of PNGase F (New England BioLabs), and labelled with 2-AB (Ludger). The labelled fetuin 2-AB glycan pool was then incubated at 37°C for 16 h in the presence of 80 μg·ml^−1^ EndoS_2_ in PBS to determine the activity of EndoS_2_ on free N-glycans. The resulting EndoS_2_-digested glycan pool was then relabelled with 2-AB. Each glycan preparation was separated using a 1.7 μm BEH glycan column (2.1 mm×150 mm, Waters) and analysed by UHPLC–FLD–MS using a Waters ACQUITY UPLC® H-Class Bio with fluorescence detection coupled to a Waters Xevo G2-S Q-ToF mass spectrometer. The column temperature was 40°C with a flow rate of 0.4 ml·min^−1^ using a linear gradient of 50 mM ammonium formate (pH 4.4) against acetonitrile with ammonium formate increasing from 30% to 47% over a 32 min period. Fluorescence detection was achieved using excitation and emission wavelengths of 330 nm and 420 nm respectively. Eluting glycans were detected in positive mode with the following settings: cone voltage of 80 V, capillary voltage of 3.0 kV, source temperature of 120°C, desolvation temperature of 300°C, and desolvation gas flow of 800 l·h^−1^. Mass data were acquired using sensitivity mode with a mass range of 750 *m*/*z* to 2000 *m*/*z* with a 1.0 s scan time. Both LC–FLD and LC–MS data were acquired and processed using Waters UNIFI version 1.6.

## RESULTS

### Identification of EndoS_2_ from GAS serotype M49

**Figure 1:**
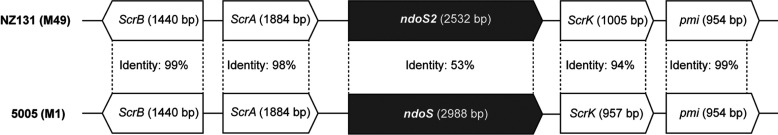
Genetic context analysis of *ndoS* and *ndoS2* The genetic context of *ndoS2* (from NZ131/M49) and *ndoS* (from MGAS5005/M1) was analysed by aligning and comparing the identity of the sequences in MacVector.

**Figure 2:**
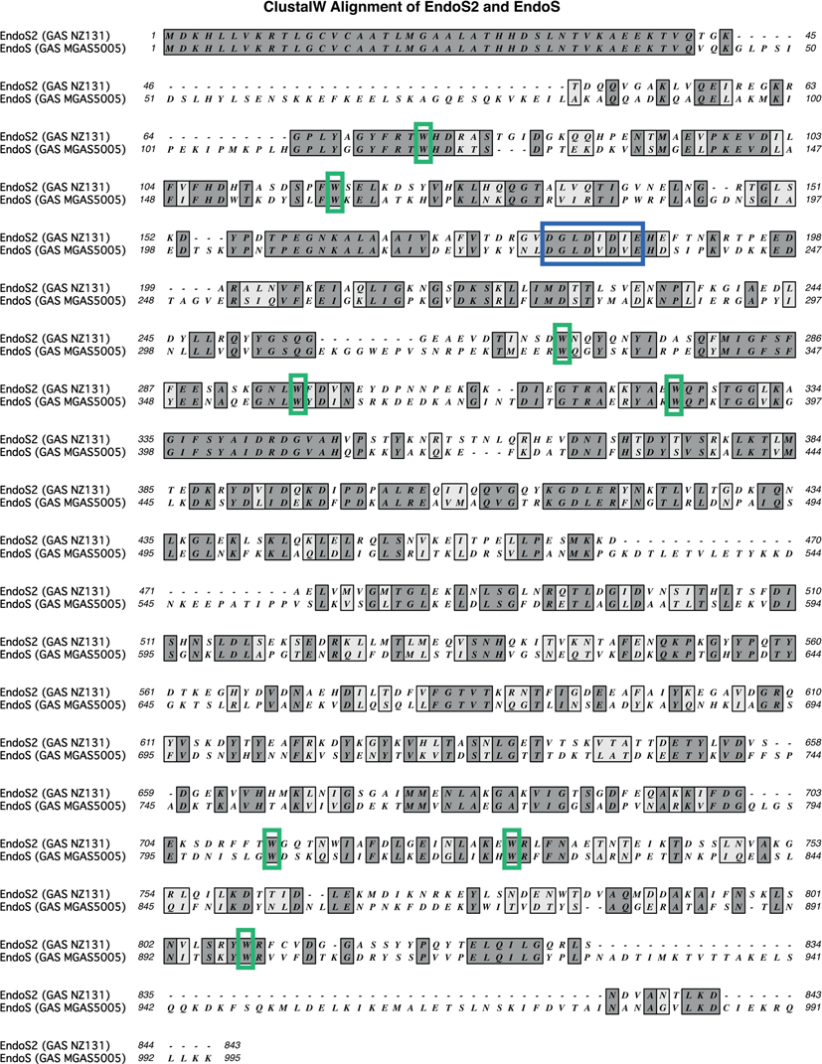
ClustalW alignment of EndoS_2_ and EndoS EndoS_2_ from GAS strain NZ131 and EndoS from GAS strain MGAS5005 was aligned using ClustalW. Depicted in blue is the GH18 active site (DXXDXDXE) and in green are conserved tryptophan residues.

In the sequenced genome of GAS strain NZ131 (serotype M49), we identified *ndoS2*, a gene harbouring a GH18 domain [6,24]. *ndoS2* from GAS serotype M49 was found in the same genetic context as *ndoS* from GAS serotype M1, but showed only 53% nucleotide identity with *ndoS* (Figure 1). The surrounding genes, i.e. *scrb, scra, scrk* and *pmi*, showed a high degree of nucleotide identity when comparing the chromosomal context between strain NZ131 and serotype M1 strain MGAS5005 (Figure 1). One genome of serotype M49 is available to the public (NZ131, GenBank® accession number NC_011375) and therefore *ndoS2* was sequenced in five M49 strains of different origin and isolation year (3487-05, ACN49, AP49, AW1 and AW2). The comparison revealed 100% identity of *ndoS2* in the five selected strains compared with *ndoS2* found in NZ131. The *ndoS2* sequences have been submitted to GenBank® (Supplementary Table S1). The deduced amino acid sequence of EndoS_2_ and EndoS revealed 37% identity when aligned using ClustalW (Figure 2). The signal peptide was conserved, but three major sections of the EndoS amino acid sequence were lacking in EndoS_2_; at positions 45–83, 535–561 and 933–986, gaps can be seen in the alignment. A comparison of the active site of EndoS_2_ and EndoS revealed the GH18 motif (DXXDXDXE) with glutamate at position 186 as the catalytic amino acid to be conserved (Figure 2). Specific tryptophan residues have previously been shown to be important for the enzymatic activity of EndoS, and when EndoS_2_ was aligned and compared with EndoS, tryptophan residues at positions 121, 164, 332, 361, 391, 809, 828 and 907 were found to be conserved [10–13].

**Figure 3:**
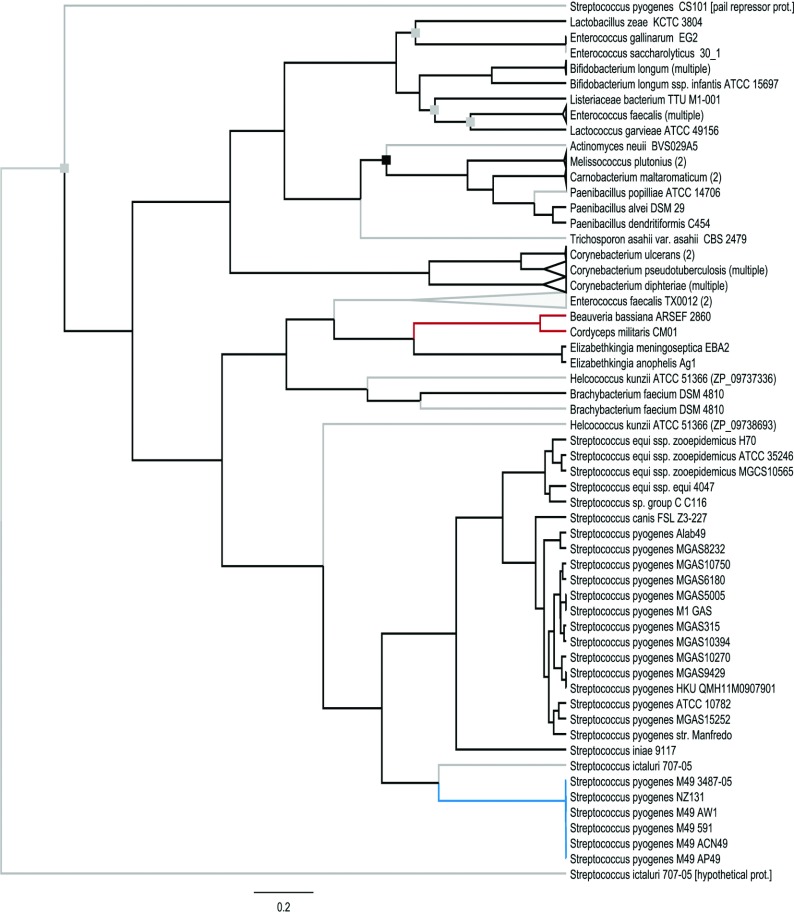
Phylogenetic reconstruction of the protein-specific tree for EndoS-like proteins, based on 1099 amino acids All internal nodes were supported by a Bayesian posterior probability (PP) of 0.99–1.0 except for nodes highlighted with grey squares (0.80<PP<0.95) or black squares (PP<0.80). The scale bar indicates genetic distance. Non-verified hypothetical proteins predicted from gene models are indicated with grey branches, the EndoS_2_ clade is highlighted in blue and the fungal clade is highlighted in red. For accession numbers, see Supplementary Table S3 at http://www.biochemj.org/bj/455/bj4550107add.htm.

In order to evaluate the evolutionary history of EndoS_2_, we reconstructed a protein specific phylogenetic tree, using BEAST version 1.7.4, on 101 protein sequences selected with the BLASTP algorithm on EndoS_2_. EndoS_2_ (depicted in blue) was found to be unique to GAS serotype M49 and relatively different from EndoS found in other serotypes of GAS as well as EndoS-like proteins in other *Streptococcus* species (Figure 3). This can be contrasted with the taxonomic phylogeny of the 16S rRNA sequences (Supplementary Figure S1 at http://www.biochemj.org/bj/455/bj4550107add.htm).

### EndoS_2_ hydrolyses the N-linked glycan on the heavy chain of IgG

**Figure 4:**
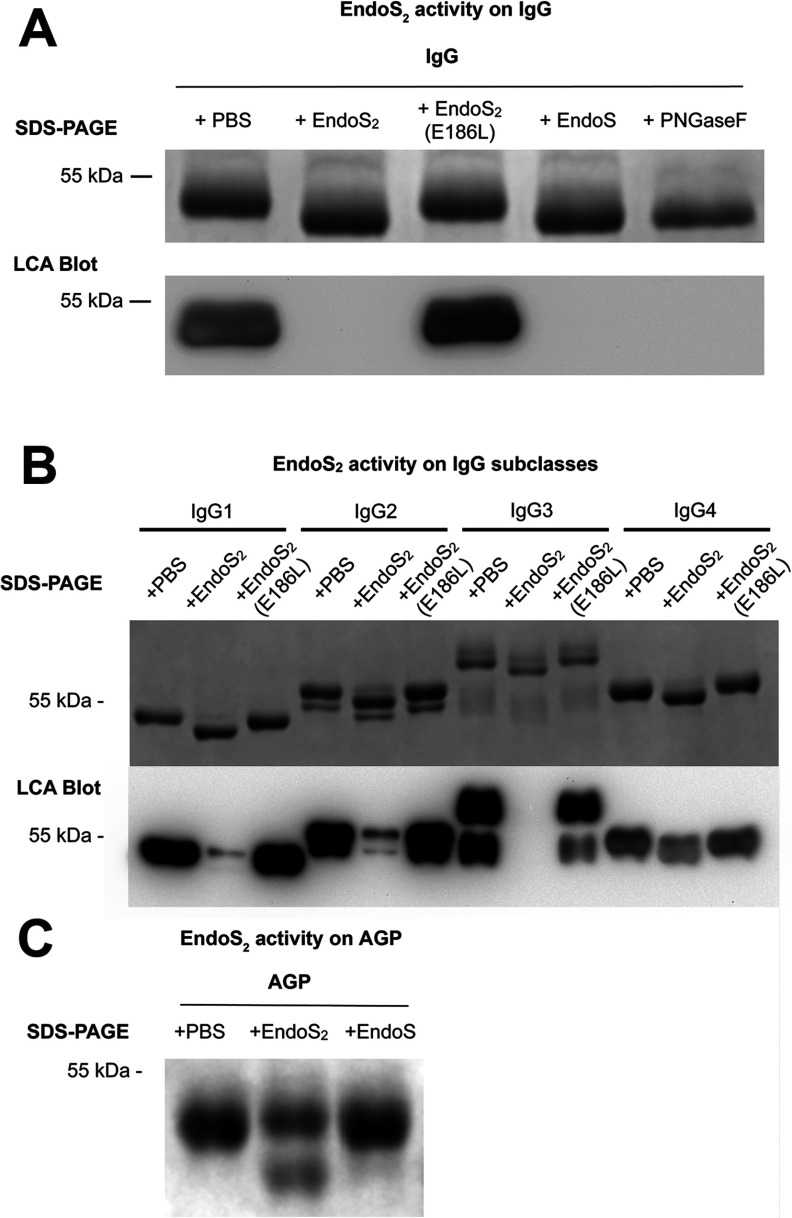
Activity of EndoS_2_ on IgG, IgG subclasses and AGP (**A**) Human serum IgG was incubated with recombinant EndoS_2_, EndoS_2_(E186L), EndoS, PNGase F or PBS at 37°C in PBS overnight and analysed by SDS/PAGE (10% gel) and a subsequent LCA blot. The gel and lectin blot shows the γ heavy chain of IgG at 50 kDa. (**B**) Human subclasses of IgG, IgG_1–4_, were incubated with recombinant EndoS_2_, EndoS_2_(E186L) or PBS in PBS at 37°C overnight and analysed by SDS/PAGE (10% gel) and a subsequent LCA lectin blot. (**C**) AGP was incubated with recombinant EndoS_2_, EndoS or PBS at 37°C in PBS overnight and analysed by SDS/PAGE (10% gel).

**Figure 5:**
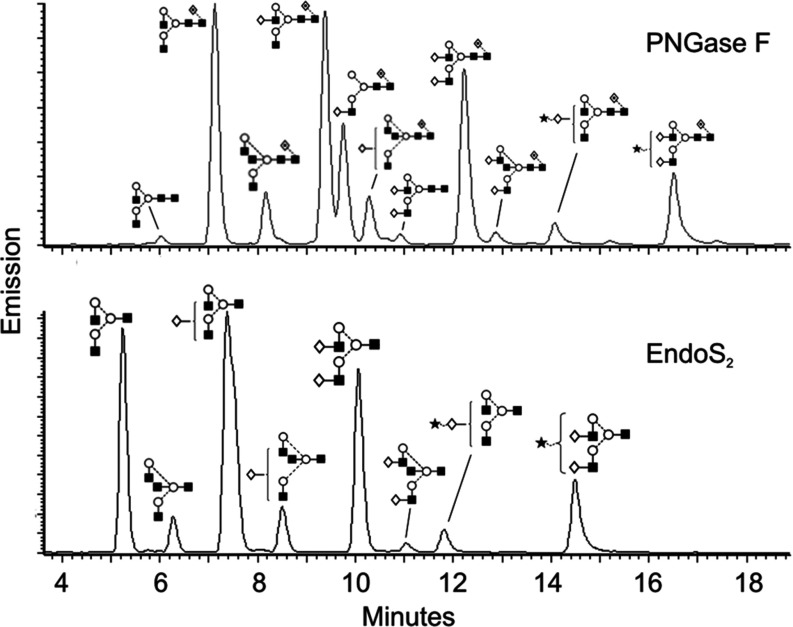
Glycan fluorescent profiles from human IgG released by EndoS_2_ and PNGase F HILIC–FLD–MS of 2-AB-labelled glycans released from human serum IgG by EndoS_2_ (**A**) and PNGase F (**B**) respectively. Identified glycan structures are presented using the Oxford glycan nomenclature [45].

Previous work has concluded that EndoS hydrolyses the N-linked glycan on IgG [10,13]. Although the enzymes are different, we tested whether IgG is a substrate for EndoS_2_. A comparison of the hydrolysis of the N-linked glycan on the heavy chain of IgG was carried out using EndoS_2_, EndoS and PNGase F as positive control [14,30]. PNGase F from *Elizabethkingia meningoseptica* cleaves between the GlcNAc and the asparagine residue of N-linked glycans, whereas EndoS cleaves between the two GlcNAc moieties in the chitobiose core of N-linked glycans [10,11]. EndoS_2_ was mutated in the active site through site-directed mutagenesis where the catalytically active glutamate residue was mutated to leucine, creating the enzyme EndoS_2_(E186L). EndoS_2_, EndoS_2_(E186L), EndoS and PNGase F were incubated with human IgG in PBS at 37°C overnight, and analysed by SDS/PAGE and a subsequent LCA (recognizing α-linked mannose) lectin blot (Figure 4A). The gel shows a ~4 kDa shift of the heavy chain of IgG and a corresponding lack of LCA lectin signal when incubated with EndoS_2_, EndoS or PNGase F, but not with EndoS_2_(E186L) or PBS (Figure 4A). This result indicates that EndoS_2_ hydrolyses the N-linked glycan on the heavy chain of IgG and confirms the glutamate residue at position 186 of EndoS_2_ to be the catalytically active amino acid. To evaluate enzymatic activity of EndoS_2_ on the subclasses of IgG, recombinant EndoS_2_ and EndoS_2_(E186L) were incubated with human IgG subclasses 1–4 and showed activity on all four human subclasses as analysed by SDS/PAGE and LCA lectin blot (Figure 4B). The glycan-hydrolysing activity of EndoS_2_ on animal IgG was found for the following species: mouse, rat, monkey, sheep, goat, cow and horse. To investigate glycan specificity of EndoS_2_, the composition of the released glycans from pooled human serum IgG was analysed by HILIC (hydrophilic interaction liquid chromatography)–UHPLC–FLD–MS and compared with the glycan profile of IgG generated by PNGase F (Figure 5). The HILIC–UHPLC–FLD–MS revealed EndoS_2_ to cleave between the two GlcNAc residues in the chitobiose core of the N-linked glycan and thus leaving a single GlcNAc residue with or without α(1,6)-linked fucose attached to the protein backbone. All peaks present in the PNGase F chromatogram could be found in the glycan profile of IgG released by EndoS_2_ with the difference of one GlcNAc with or without α(1,6)-linked fucose.

### EndoS_2_ releases biantennary and sialylated glycans on AGP

**Figure 6:**
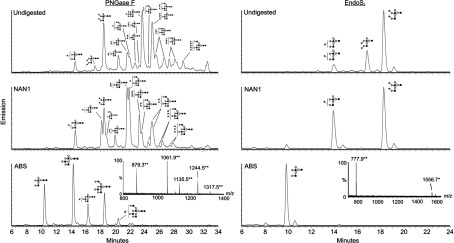
Glycan fluorescent profiles from human AGP released by PNGase F and EndoS_2_ HILIC–FLD–MS of 2-AB-labelled glycans released from human AGP by PNGase F (left) and EndoS_2_ (right) respectively. 2-AB-labelled glycans were digested further with NAN1 and ABS, and subsequent bi-, tri- and tetra-antennary structures are indicated using the Oxford glycan nomenclature [45]. Ions were detected as [*M*−2H]^2−^ (**) and [*M*−H]^−^ (*) species.

AGP, also known as orosomucoid, is a 41–45 kDa human plasma glycoprotein, a major positive acute-phase protein, up-regulated severalfold during inflammation and a member of the lipocalin family [11,31]. The immunomodulatory effects of AGP is linked to the carbohydrate composition of the five N-linked glycans (Asn^33^, Asn^56^, Asn^72^, Asn^93^ and Asn^103^) that make up 45% of the molecular mass [15,31]. When incubating AGP with recombinant EndoS_2_, subsequent SDS/PAGE revealed a new band at ~38 kDa and a decrease in the intensity of the band at 45 kDa (Figure 4C). No activity was detected with EndoS or PBS in the same assay (Figure 4C). To elucidate the enzymatic activity of EndoS_2_ on AGP in detail, we analysed the glycans released from AGP by EndoS_2_ using HILIC–UHPLC–FLD–MS and exoglycosidase arrays in UHPLC (Figure 6). The sequence, composition and linkage specificities of all glycoforms of AGP released by PNGase F were determined in the same way to serve as control. EndoS_2_ was found to cleave only biantennary and sialylated structures of AGP, whereas the glycan profile from PNGase F contained sialylated bi-, tri- and tetra-antennary structures with or without outer arm fucosylation. The cleavage site of EndoS_2_ was confirmed to be between the two GlcNAcs in the chitobiose core of the glycan. The glycan profiles were digested with NAN1 to remove α(2,3)-linked sialic acids and ABS to remove α(2,3)-, α(2,6)- and α(2,8)-linked sialic acid residues. The resulting bi-, tri- and tetra-antennary structures from AGP were identified as *M*−2H^2−^ ions (*m*/*z* 879.3, 1061.9, 1135.5, 1244.5 and 1317.5). Furthermore, glycans were digested with linkage-specific exoglycosidases to verify the presence of outer arm fucosylation. These enzymes were BTG, BKF and AMF (Supplementary Figure S2 at http://www.biochemj.org/bj/455/bj4550107add.htm). AMF digestion removed α(1,3) non-reducing terminal fucose linked to galactose residues and not core α(1,6)-fucose. BKF treatment, which is specific for core α(1,6)-linked fucose residues, did not result in glycan digest products.

### EndoS_2_ is specific for IgG and AGP and not a general chitinase

**Figure 7:**
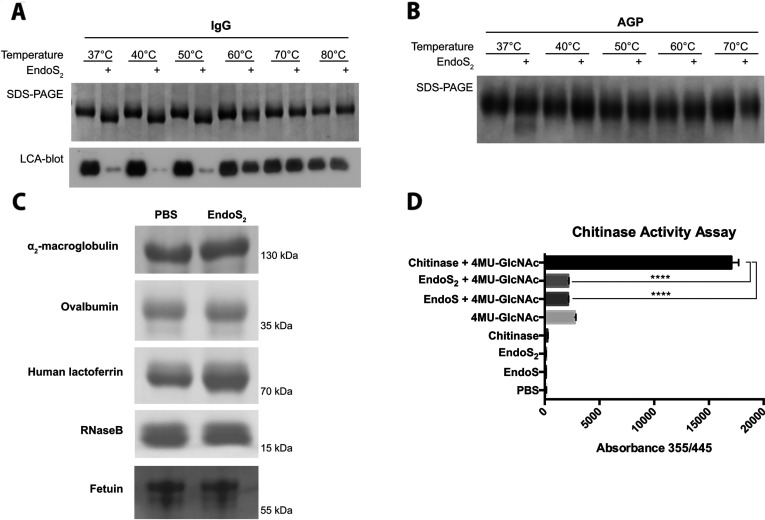
Activity of EndoS_2_ on native and denatured IgG and AGP, other glycoproteins and chitinase assay (**A**) IgG was incubated at temperatures ranging from 37 to 80°C for 30 min followed by incubation with EndoS_2_ at 37°C for 2 h and analysis by SDS/PAGE and LCA lectin blot. (**B**) AGP was incubated at 37–70°C followed by incubation with EndoS_2_ at 37°C for 2 h and analysis by SDS/PAGE. (**C**) EndoS_2_ was incubated with α_2_-macroglobulin, ovalbumin, human lactoferrin, RNase B and fetuin at 37°C overnight and analysed by SDS/PAGE. (**D**) EndoS_2_, EndoS and a chitinase from *S. griseus* was incubated with the fluorescent substrate 4MU-GlcNAc for 1 h and fluorescence was measured at 355/445 nm. The experiments were carried out using five replicates and results are means±S.D. The response in absorbance was analysed statistically by an unpaired Student's *t* test, where differences was considered significant if *P*<0.05. ****P<0.001.

Previous work on EndoS has shown that the enzyme is specific for the native form of IgG [14]. To test whether this is valid for EndoS_2_, IgG and AGP were incubated at temperatures ranging from 37 to 80°C or 37 to 70°C for 30 min before the addition of EndoS_2_ or PBS and a 2 h incubation at 37°C. SDS/PAGE analysis revealed a shift of IgG incubated at 37–50°C and loss of signal was seen in a corresponding LCA lectin blot, whereas only partial shift could be seen at 60°C and no shift and intact LCA signal at temperatures 70°C and 80°C (Figure 7A). Glycans from AGP were hydrolysed at 37°C, but not at 40–70°C (Figure 7B). The activity of EndoS_2_ was tested further on a range of glycoproteins, i.e. α_2_-macroglobulin, ovalbumin, lactoferrin, RNase B and fetuin, but no activity could be detected (Figure 7C). To study whether EndoS_2_ shows general chitinase activity, we employed the substrate 4MU-GlcNAc, which fluoresces when cleaved, to compare the enzymatic activity of EndoS_2_ and EndoS with that of a chitinase from *S. griseus*. The results indicate that neither EndoS_2_ nor EndoS has a general chitinase activity compared with the positive control (Figure 7D).

### EndoS_2_ hydrolyses free biantennary glycans

**Figure 8:**
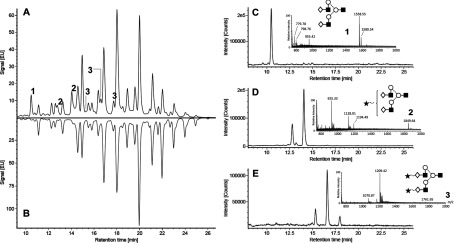
EndoS_2_ hydrolyses biantennary free glycans Bovine fetuin N-glycans were released with PNGase F, labelled with 2-AB, and analysed by HILIC–UHPLC–FLD–MS. Released N-glycans were digested further with EndoS_2_ to determine enzymatic activity on free glycans. Comparison of the fluorescent chromatograms of glycans after PNGase F (**B**) and subsequent EndoS_2_ digestion (**A**) identified three unique peaks (labelled 1, 2 and 3). These peaks correspond to three isomeric structures (A2G2, A2G2S1 and A2G2S2) and were detected primarily as *m*/*z* 1558.55 [*M*+H]^+^, 925.33 [*M*+2H]^2+^ and 1070.87 [*M*+2H]^2+^ ions respectively (**C**)–(**E**). Extracted ion chromatograms of A2G2S1 and A2G2S2 precursor ions identified structural isomers, presumably from variation in sialic acid linkages.

Following the findings of the specificity of EndoS_2_, we asked the question whether the enzyme is substrate-specific and/or has glycoform selectivity. To test this, we analysed the activity of EndoS_2_ on free glycans. All glycoforms from bovine fetuin were released using PNGase F and were 2-AB-labelled; in a secondary reaction, the free glycans were incubated with EndoS_2_, relabelled with 2-AB and analysed using HILIC–UHPLC–FLD–MS. Three structures in the chromatogram (labelled 1, 2 and 3) were modified by EndoS_2_ compared with the PNGase F glycan pool (Figure 8A and 8B). The *m*/*z* of the [*M*+H]^+^ ions of these structures were identified and revealed structures for A2G2 (1558.5513), A2G2S1 (925.3294) and A2G2S2 (1070.8700) less one GlcNAc residue (Figures 8C–8E). The results indicate that EndoS_2_ specifically hydrolyses free biantennary glycoforms with or without terminal sialylation.

### Expression of EndoS_2_ is linked to carbohydrate utilization

**Figure 9:**
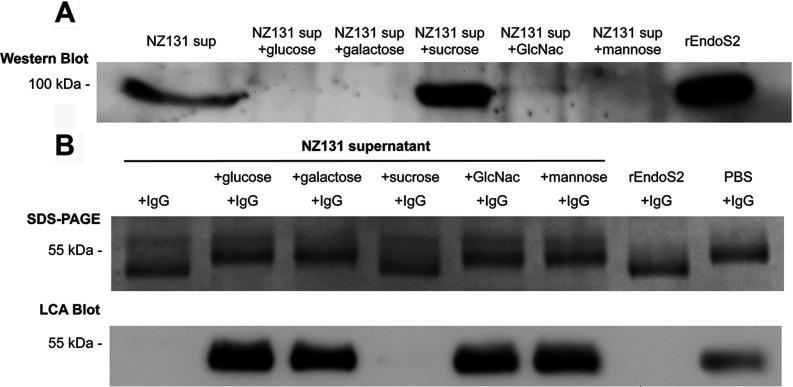
EndoS_2_ expression and activity in GAS strain NZ131 (**A**) Western immunoblot showing EndoS_2_ in bacterial supernatants with or without added carbohydrates. rEndoS_2_ was used as a positive control. (**B**) Lectin blot analysis of human IgG incubated with bacterial supernatants with or without carbohydrates, or rEndoS_2_ as a positive control.

To confirm the findings with recombinant EndoS_2_, the expression levels and enzymatic activity of EndoS_2_ were analysed in GAS supernatants. The expression of EndoS in serotype M1 of GAS is maximized in the nutrient-poor CM [10,16–21]. Therefore expression of EndoS_2_ was analysed by Western blotting of 16 h bacterial supernatants grown in CM. However, EndoS_2_ could only be detected in the supernatant when GAS was cultured in 50% diluted CM, when the bacteria are starved (Figure 9A). In a subsequent functional assay incubating IgG with the bacterial supernatant, loss of the N-linked glycan on IgG was visualized as a 4 kDa shift of the heavy chain on the electrophoresis gel and corresponding lack of signal in the LCA lectin blot (Figure 9B). This experiment confirmed the activity of the native protein in the bacterial supernatant. The expression of EndoS_2_ in poor medium led us to believe that the expression of EndoS_2_ was linked to the carbohydrate utilization of the bacteria [22,23,32]. To address this, a selection of carbohydrates was added to the bacterial culture medium and the expression of EndoS_2_ was studied. Adding glucose, galactose, GlcNAc or mannose to 50% CM inhibited EndoS_2_ expression, whereas additional sucrose increased the amount of EndoS_2_ in the supernatant (Figure 9A).

## DISCUSSION

The study of bacterial glycosidases has emerged as a field at the intersection of microbial pathogenesis and glycobiology. By studying the mechanisms by which bacteria interfere with host glycosylation, new insight can be gained into both bacterial pathogenesis and the impact of glycosylation of the immune system. Interfering with the glycosylation of the host defence is widespread among pathogenic bacteria for modulation of the functions of the immune system or as a way of utilizing the glycans of glycoproteins as nutrients [24,33].

For example, *Enterococcus faecalis*, a Gram-positive gut bacterium and opportunist, secretes EndoE, an endoglycosidase with activity on the Fc-glycan on IgG and on the glycoprotein RNase B that promotes bacterial growth when nutrients are scarce [24,34]. The endoglycosidases EndoF_1–3_ from *E. meningoseptica* and EndoH from *Streptomyces plicatus* has been shown to be glycan-specific: high-mannose and hybrid oligosaccharides are cleaved by EndoF_1_ and EndoH, whereas complex biantennary and bi- and tri-antennary glycans are released by EndoF_2_ and EndoF_3_ respectively [24,25,35–38]. An N-glycan deglycosylation complex in *Capnocytophaga canimorsus* has been found to cleave off N-linked glycans from IgG and to transport the glycans across the cell membrane for glycan catabolism [24,25,39]. *S. pneumoniae* has three surface-anchored exoglycosidases that work in concert to remove sialic acid, galactose and GlcNAc on human glycoproteins [40]. GAS EndoS was thought to be conserved throughout the GAS serotypes, and only minor variations are found when comparing *ndoS* among the sequenced GAS strains. It was therefore surprising to find that GAS strain NZ131 harboured *ndoS2*, with 53% identity with *ndoS.* The sequenced *ndoS2* in five different M49 strains revealed high identity, arguing that this gene is conserved throughout the serotype.

In the phylogenetic protein tree, the EndoS_2_ group is relatively different from EndoS in both *S. pyogenes* and in other *Streptococcus* species (but it groups with a hypothetical protein found in *Streptococcus ictaluri*). In general, the patterns are not uniform: whereas within-species or within-genus similarity of the EndoS-like proteins is high for some taxonomic groups (e.g. *Bifidobacterium longum*), there is also considerable within-genus variation in *Corynebacterium* (Figure 3). Strikingly, EndoS and EndoS_2_ from *Streptococcus* are more closely related to EndoS-like proteins of the fungi *Cordyceps militaris* and *Beauveria bassiana* than to EndoS-like proteins of bacteria such as *Melissococcus*, *Corynebacterium* and *Lactobacillus* (Figure 3), in sharp contrast with the taxonomic relationships (Supplementary Figure S1). Notably, some taxa are paraphyletic in the EndoS-like protein phylogeny, to which could possibly be ascribed the inclusion of non-verified hypothetical proteins. However, the biologically verified EndoS-like proteins of *Enterococcus gallinarum* and *E. faecalis* do not form a monophyletic clade (Figure 3). In all, this picture indicates the occurrence of horizontal gene transfer of *ndoS*-like genes. Even though no known proteins were found to be closely related to EndoS_2_, the differentiation from *S. pyogenes* EndoS and the high degree of similarity between serotype M49 and other *S. pyogenes* strains combined with the conserved genetic context points towards horizontal gene transfer of *ndoS2* into serotype M49. The strain NZ131 also has an unusually high frequency of transformation, and horizontal gene transfer has been described on several places in the genome [24]. The alternative interpretation, that a particularly strong directional selection on the ancestral *ndoS* gene in serotype M49 resulted in *ndoS2*, seems less plausible.

The active site and tryptophan residues important for activity in EndoS were found to be conserved in EndoS_2_ even though the proteins are only 37% identical [13]. Despite this substantial difference in amino acid sequences, EndoS_2_ hydrolysed the glycan on IgG in a similar fashion to EndoS. In the chromatograms comparing the glycan profile of IgG generated by EndoS_2_ and PNGase F (Figure 5), a shift was observed that could be explained by the site of action. PNGase F is an amidase that cleaves between the asparagine residue and the first GlcNAc residue of the glycan, whereas EndoS_2_ cleaves after the first GlcNAc and thus leaves one GlcNAc with or without fucose attached to the protein backbone. Owing to lack of one reducing end GlcNAc in the EndoS_2_ glycan profile, there is a loss in resolution, which explains why the A2G1 peak could not be separated in the EndoS_2_ chromatogram, but can be seen as two separate peaks in the PNGase F profile. It has been argued previously that EndoS does not cleave bisecting glycans [41,42]. From the LC–MS data of the present study, we argue that EndoS_2_ cleaves all glycoforms present of human serum IgG, including bisecting glycans, since all peaks present in the PNGase F glycan profile could be found in the EndoS_2_ profile (Figure 5).

A striking difference between EndoS_2_ and EndoS was found when incubated with the human acute-phase protein AGP. The observed activity of EndoS_2_ was confirmed with LC–MS and revealed that EndoS_2_ specifically releases biantennary and sialylated structures of AGP (Figure 6). Again, the peaks annotated in the EndoS_2_ profile could be found in the PNGase F release with the difference of one GlcNAc residue. It is clear that EndoS_2_ only releases a fraction of the glycans present on AGP. EndoS_2_ does not cleave tri- and tetra-antennary glycans, with or without outer arm fucosylation, although they are present in great numbers on AGP.

The activity on IgG and AGP raised several questions regarding the specificity of EndoS_2_. To answer these, we tested the activity of EndoS_2_ on heat-denatured IgG and AGP, on other glycoproteins, in a chitinase assay and on a pool of free N-glycans. EndoS_2_ was only active on native IgG and AGP and we draw the conclusion that EndoS_2_ requires a protein–protein interaction with its substrates for glycan hydrolysis to occur. The activity of EndoS_2_ on AGP may be the result of reduced protein recognition, since early studies indicate sequence homology between IgG and AGP [43]. On glycoproteins with a completely different fold, we detected no activity with similar assays to the activity on IgG and AGP detected. It was therefore not surprising to find that EndoS_2_ had no general chitinase activity compared with a chitinase from *S. griseus*. Taken together, these data indicate that EndoS_2_ specifically interacts with protein folds including IgG and AGP. Furthermore, we dissected the glycoform specificity of EndoS_2_ by incubating the enzyme with the N-glycan pool from fetuin released by PNGase F and showed that EndoS_2_ hydrolysed only free biantennary structures with or without terminal sialylation. No bisecting glycans are present on fetuin, which explains why such structures are not present in the chromatograms. On the basis of our findings, we believe that EndoS_2_ is both site- and glycoform-specific which is a unique property of an endoglycosidase.

The hydrolysis of the glycan of IgG has been shown to have major consequences on the effector functions of the antibody by modulating the binding to FcγR [12]. Since EndoS and EndoS_2_ have similar hydrolysing activity on the glycan of IgG, both enzymes are expected to affect the functionality of this antibody. The functional consequence for AGP when biantennary sialylated glycans are cleaved off is unknown and lies beyond the scope of the present study.

The expression of EndoS_2_ was found to depend on the availability of carbohydrates in the bacterial culture medium. C-medium is a poor medium for GAS and expression of EndoS_2_ could only be detected when GAS was grown in 50% diluted C-medium. Incubating the supernatants with IgG confirmed the previous work carried out with recombinant EndoS_2_ and a clear correlation between expression of EndoS_2_ and hydrolysis of the Fc-glycan on IgG confirmed this. The genes *scrb*, *scra* and *scrk*, surrounding *ndoS2*, are part of a sucrose utilization operon and this could explain the increase of EndoS_2_ expression when sucrose was added to the culture medium. The presence of glucose, galactose, GlcNAc or mannose completely inhibited expression of EndoS_2_ indicating that this enzyme is tightly regulated by a mechanism sensitive to the presence of carbohydrates. Research has indicated that the virulence of GAS is linked to the utilization of available carbohydrates via CcpA (catabolite control protein A), but in the present study of EndoS_2_, we can only hypothesize that CcpA is involved in the regulation mechanism [32]. This indicates that in the infection scenario, EndoS_2_ is strictly regulated and that the enzyme is used in an environment where nutrition is scarce, e.g. the human skin. This indicates further that the virulence of GAS is linked to the utilization of complex carbohydrates [44].

The present study shows that the endoglycosidase EndoS_2_ is conserved and uniquely present in GAS serotype M49. We show that EndoS_2_ hydrolysed all glycoforms on human serum IgG and biantennary and sialylated glycans on AGP. EndoS_2_ is secreted by GAS during starvation and the expression is linked to the carbohydrate composition of the culture medium. The enzymatic activity on two key players of the immune system argues that EndoS_2_ has a role in immunomodulation of the host that could potentially be linked to the pathogenesis of GAS serotype M49 infections.

## Online data

Supplementary data
