# Supplementary material for: EndoS2 is a unique and conserved enzyme of serotype M49 group A Streptococcus that hydrolyses N-linked glycans on IgG and α1-acid glycoprotein
Source: Biochem J. 2013 Sep 13;455(Pt 1):107–18. doi: 10.1042/BJ20130126 (PMC3778708; doi:10.1042/BJ20130126)
Supplement: Supplementary data [file bj4550107add.pdf]

Biochem. J. (2013) **455**, 107–118 (Printed in Great Britain) doi:10.1042/BJ20130126

SUPPLEMENTARY ONLINE DATA

EndoS$_2$ is a unique and conserved enzyme of serotype M49 group A *Streptococcus* that hydrolyses N-linked glycans on IgG and α_1-acid glycoprotein

Jonathan SJÖGREN*[1,2] , Weston B. STRUWE†[3], Eoin F. J. COSGRAVE†[4], Pauline M. RUDD†, Martin STERVANDER‡, Maria ALLHORN*[2], Andrew HOLLANDS§||[2], Victor NIZET*||¶[2] and Mattias COLLIN*[2]

*Department of Clinical Sciences, Division of Infection Medicine, Lund University, Biomedical Center B14, SE-22184 Lund, Sweden, †The National Institute for Bioprocess Research and Training, NIBRT, University College Dublin, Fosters Avenue, Mount Merrion, Blackrock, Co. Dublin, Ireland, ‡Molecular Ecology and Evolution Laboratory, Department of Biology, Lund University, Ecology Building, SE-223 62 Lund, Sweden, §School of Biological Sciences, University of Wollongong, Wollongong, NSW 2522, Australia, ||Department of Pediatrics, University of California San Diego, 9500 Gilman Drive, Mail Code 0687, La Jolla, CA 92093, U.S.A., and ¶Skaggs School of Pharmacy and Pharmaceutical Sciences, University of California San Diego, 9500 Gilman Drive, Mail Code 0687, La Jolla, CA 92093, U.S.A.

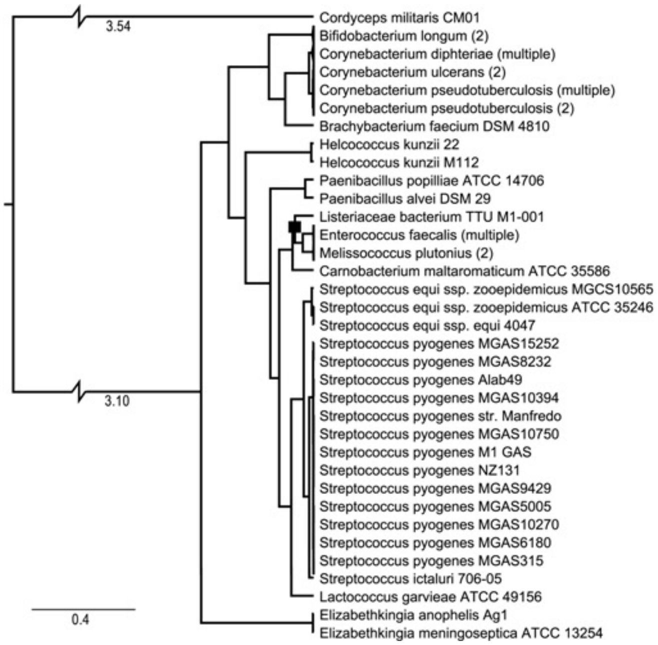

Figure S1 Phylogenetic reconstruction of 16S rRNA sequences, for inference of taxonomic relationship between taxa of the EndoS-like protein phylogeny, based on 1576 nucleotides

All internal nodes were supported by a Bayesian posterior probability (PP) of 0.99–1.0 except for the grouping of the Listeriaceae family bacterium, highlighted with a black squares signifying PP<0.80. The scale bar indicates genetic distance. Note that the branches connecting the ingroup and outgroup are truncated owing to the large taxonomic difference (genetic distances stated below branches). For accession numbers, see Table S3.

[1] To whom correspondence should be addressed (email jonathan.sjogren@med.lu.se).
[2] Patents for the use of EndoS$_2$ have been applied for by Genovis AB. Jonathan Sjögren, Andrew Hollands, Victor Nizet, Maria Allhorn and Mattias Collin are listed as inventors on the application that is pending.
[3] Present address: Chemistry Research Laboratory, Department of Chemistry, University of Oxford, Oxford OX1 3TA, U.K.
[4] Present address: Waters Corporation, 34 Maple Street, Milford, MA 01757, U.S.A.
The sequences of *Streptococcus pyogenes ndoS2* will appear in the DDBJ, EMBL, GenBank® and GSDB Nucleotide Sequence Databases under accession numbers KC155346 (strain 3487-05), KC155348 (strain AP49), KC155347 (strain ACN49), KC155349 (strain AW1) and KC155350 (strain AW2).

© 2013 The Author(s)
The author(s) has paid for this article to be freely available under the terms of the Creative Commons Attribution Licence (CC-BY) (http://creativecommons.org/licenses/by/3.0/) which permits unrestricted use, distribution and reproduction in any medium, provided the original work is properly cited.

Biochemical Journal www.biochemj.org

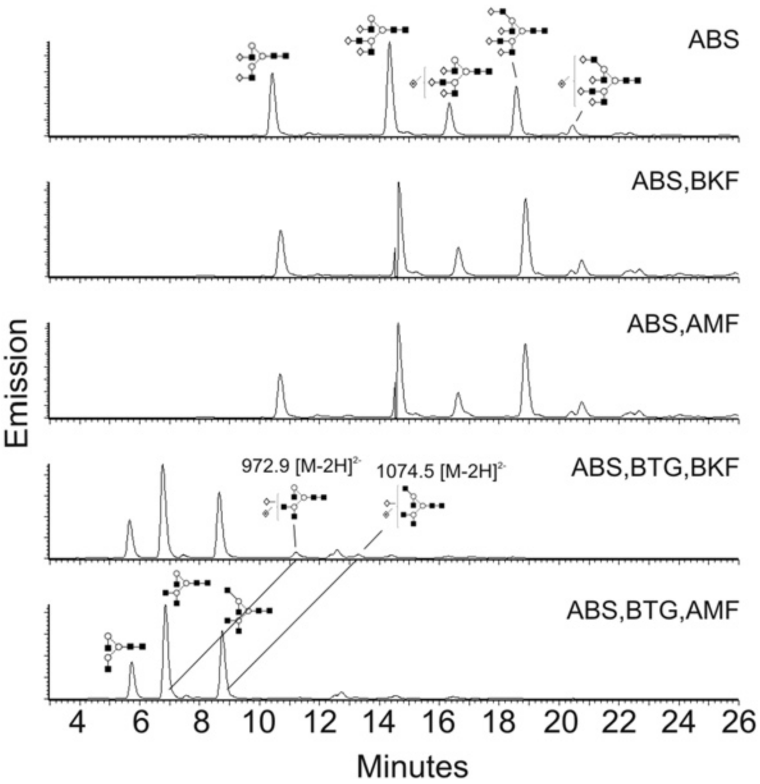

Figure S2 HILIC–FLD–MS and exoglycosidase sequencing of AGP N-glycans

The fucose linkage position was confirmed as α(1,3) to galactose residues on tri- and tetra-antennary glycans and were detected as $[M-2H]^{2-}$ ions 972.9 and 1074.5.

Table S1 *E. coli* and group A streptococcal strains used in the present study

Strains of *E. coli* and *S. pyogenes* with accession numbers. Also indicated is the GenBank® accession number for *ndoS2* sequences. N/A, not applicable.

Strain	Source/accession number	*ndoS2* accession number
E. coli		
Top10	Invitrogen	N/A
BL21 pLysS	Invitrogen	N/A
GAS (serotype)		
NZ131 (M49)	ASM1812v1	ACI61688
3487-05 (M49)	*S. pyogenes*, serotype M49	KC155346
ACN49 (M49)	Strain 3274-98 from Center for Disease Control and prevention. A gift from Actinova	KC155347
AP49 (M49)	*S. pyogenes* strain collection, Institute of Hygiene and Epidemiology, Prague, Czech Republic	KC155348
AW1 (M49)	Nephritis strain H 9449 B from the collection of L. Wannamaker	KC155349
AW2 (M49)	Strain H 5424 from the collection of L. Wannamaker	KC155350

© 2013 The Author(s)
The author(s) has paid for this article to be freely available under the terms of the Creative Commons Attribution Licence (CC-BY) (http://creativecommons.org/licenses/by/3.0/) which permits unrestricted use, distribution and reproduction in any medium, provided the original work is properly cited.

Table S2 Plasmids and primers used in the present study

Plasmids and primers used for recombinant expression and sequencing.

(a) Plasmids

Name	Source
pGEX-5X-3	GE Healthcare
pGEX-ndoS2	The present study
pGEX-ndoS2(E-L)	The present study
pGEX-ndoS	[1]
pCR2.1	Invitrogen

(b) Primers

Name	Sequence (5′→3′)
ndoS2-out-R	GCGCCACTTTCTGGTAGTCTAAC
Seq38-R	TTTGGGACCTCAGCCATAG
Seq42-R	GGATTGTTAGGGTCGTATTCG
Seq54-R	TTTAGCCCCTTTTGCCAG
Seq15-F	CCTGAAGAAGATGCTCGTG
Seq17-F	TGATGACCGAAGACAAACG
Seq24-F	GAGACGAAGAAGCATTTGC
Seq28-F	TCACTTGGGGACAAACTAAC
ndoS2-F-BamHI	CTGTAAGGATCCAGGAGAAGACTG
ndoS2-R-XhoI	GAAACCTCGAGTCTTTGTAATCGTAGGACTT
ndoS2(E-L)-F-BamHI	CTAGATATTGATATTCTTCACGAATTTACGAAC
ndoS2(E-L)-R-XhoI	GTTCGTAAATTCGTGAAGAATATCAATATCTAG

© 2013 The Author(s)
The author(s) has paid for this article to be freely available under the terms of the Creative Commons Attribution Licence (CC-BY) (http://creativecommons.org/licenses/by/3.0/) which permits unrestricted use, distribution and reproduction in any medium, provided the original work is properly cited.

Table S3 Accession numbers for EndoS-like protein and 16S rRNA sequences

For the EndoS-like protein dataset, outgroup sequences are indicated with an asterisk. For the $EndoS_2$ sequences of the present study, see Table S1.

		GenBank® accession number	
Taxa	Strain	EndoS-like protein	16S rRNA
Actinomyces neuii	BVS029A5	ZP_11040533	
Beauveria bassiana	ARSEF 2860	EJP67240	
Bifidobacterium longum subsp. *infantis*	157F	YP_004209228	AP010890
Bifidobacterium longum subsp. *infantis*	ATCC 15697	YP_002323900	AP010889
Bifidobacterium longum subsp. *longum*	44B	EIJ32132	
Bifidobacterium longum subsp. *longum*	1-6B	EIJ25186	
Brachybacterium faecium	DSM 4810	YP_003153977; YP_003155571	X91032
Carnobacterium maltaromaticum	ATCC 35586	ZP_10279268	JF749289
Carnobacterium maltaromaticum	LMA28	YP_006993899	
Chryseobacterium meningosepticum	ATCC 13254	AJ704541	
Cordyceps militaris	CM01	EGX89767	HQ585075
Corynebacterium diphtheriae	241	YP_005126330	CP003207
Corynebacterium diphtheriae	BH8	YP_005161243	CP003209
Corynebacterium diphtheriae	C7 (β)	YP_005163607	CP003210
Corynebacterium diphtheriae	CDCE 8392	YP_005134619	CP003211
Corynebacterium diphtheriae	INCA 402	YP_005128539	CP003208
Corynebacterium diphtheriae	PW8	YP_005143699	CP003216
Corynebacterium diphtheriae	31A	YP_005158899	
Corynebacterium diphtheriae bv. *intermedius* str.	NCTC 5011	EIK55328	
Corynebacterium pseudotuberculosis	31	YP_006214375	CP003421
Corynebacterium pseudotuberculosis	258	YP_006353466	CP003540
Corynebacterium pseudotuberculosis	Cp162	YP_006437976	CP003652
Corynebacterium pseudotuberculosis	FRC41	YP_003784295	CP002097
Corynebacterium pseudotuberculosis	PAT10	YP_005691331	CP002924
Corynebacterium pseudotuberculosis	X81907		
Corynebacterium pseudotuberculosis	316	YP_005304514	CP003077
Corynebacterium pseudotuberculosis	CIP 52.97	YP_005695446	
Corynebacterium ulcerans	809	YP_005711595	CP002790
Corynebacterium ulcerans	BR-AD22	YP_004630749	CP002791
Elizabethkingia anophelis	Ag1	ZP_09415888	EF426425
Elizabethkingia meningoseptica	EBA2	FLAME	
Enterococcus faecalis	ATCC 29200	ZP_04439322	ACHK01000081
Enterococcus faecalis	OG1RF	CP002621	
Enterococcus faecalis	V583	NP_813917	
Enterococcus faecalis	HER1044	AAR20477	
Enterococcus faecalis	DS5	ZP_05561082	
Enterococcus faecalis	ERV85	EJV36587	
Enterococcus faecalis	PC1.1	ZP_06746878	
Enterococcus faecalis	R508	EJV39420	
Enterococcus faecalis	R712	ZP_06630726	
Enterococcus faecalis	T1	ZP_05422239	
Enterococcus faecalis	T2	ZP_05425204	
Enterococcus faecalis	T3	ZP_05502539	
Enterococcus faecalis	T8	ZP_05560447	
Enterococcus faecalis	TX0012	EFT95260; EFT95516; EFT95520	
Enterococcus faecalis	TX0104	ZP_03950001	
Enterococcus faecalis	TX0109	ZP_07567140	
Enterococcus faecalis	TX0309B	EFU85828	
Enterococcus faecalis	TX0470	ZP_07760464	
Enterococcus faecalis	TX0855	ZP_07555326	
Enterococcus faecalis	TX0860	ZP_07559314	
Enterococcus faecalis	TX1322	ZP_04435748	
Enterococcus faecalis	TX1341	EFU12187	
Enterococcus faecalis	TX1342	EFU15396	
Enterococcus faecalis	TX1346	EFU16620	
Enterococcus faecalis	TX1467	EGG58918	
Enterococcus faecalis	TX2137	EFT38634	
Enterococcus faecalis	TX4244	EFT91060	
Enterococcus gallinarum	EG2	ZP_05648160	
Enterococcus saccharolyticus	30_1	ZP_09110633	
Helcococcus kunzii	ATCC 51366	ZP_09737336; ZP_09738693	
Helcococcus kunzii	22	NR_029237	
Helcococcus kunzii	M112	JN861738	
Lactobacillus zeae	KCTC 3804	ZP_09453558	
Lactococcus garvieae	ATCC 49156	YP_004779109	AP009332
Listeriaceae family bacterium	TTU M1-001	ZP_09890591	JQ287762
Melissococcus plutonius	ATCC 35311	YP_004455741	AP012200

© 2013 The Author(s)
The author(s) has paid for this article to be freely available under the terms of the Creative Commons Attribution Licence (CC-BY) (http://creativecommons.org/licenses/by/3.0/) which permits unrestricted use, distribution and reproduction in any medium, provided the original work is properly cited.

Table S3 Continued

		GenBank® accession number	
Taxa	Strain	EndoS-like protein	16S rRNA
Melissococcus plutonius	DAT561	YP_005320150	AP012282
Paenibacillus alvei	DSM 29	ZP_10866627	AJ320491
Paenibacillus dendritiformis	C454	ZP_09678120	
Paenibacillus popilliae	ATCC 14706	GAC41484	AB073198
Streptococcus canis	FSL Z3-227	ZP_10274531	
Streptococcus equi subsp. *equi*	4047	YP_002745815	FM204883
Streptococcus equi subsp. *zooepidemicus*	MGCS10565	YP_002122753	CP001129
Streptococcus equi subsp. *zooepidemicus*	ATCC 35246	AEJ24585	CP002904
Streptococcus equi subsp. *zooepidemicus*	H70	YP_002745127	
Streptococcus ictaluri	707-05	ZP_09127116; ZP_09126970*	
Streptococcus ictaluri	706-05	DQ462420	
Streptococcus iniae	9117	ZP_11067943	
Streptococcus pyogenes	Alab49	YP_006072662	CP003068
Streptococcus pyogenes	M1 GAS	NP_269818	AE006615
Streptococcus pyogenes	MGAS315	NP_665372	AE014074
Streptococcus pyogenes	MGAS5005	YP_282903	CP000017
Streptococcus pyogenes	MGAS6180	YP_280992	CP000056
Streptococcus pyogenes	MGAS8232	NP_607886	AE009954
Streptococcus pyogenes	MGAS9429	YP_597275	CP000259
Streptococcus pyogenes	MGAS10270	YP_599216	CP000260
Streptococcus pyogenes	MGAS10394	YP_060848	CP000003
Streptococcus pyogenes	MGAS10750	YP_603093	CP000262
Streptococcus pyogenes	MGAS15252	YP_005389405	CP003116
Streptococcus pyogenes	str. Manfredo	YP_001127900	AM295007
Streptococcus pyogenes	NZ131	YP_002286383	CP000829
Streptococcus pyogenes	ATCC 10782	ZP_07460044	
Streptococcus pyogenes	HKU QMH11M0907901	EIK41346	
Streptococcus pyogenes	M49 591	ZP_00365754	
Streptococcus pyogenes	CS101	U78969_1*	
Streptococcus sp. group C	C116	ADC53484	
Trichosporon asahii var. *asahii*	CBS 2479	EJT46459	

REFERENCE

1 Collin, M. and Olsén, A. (2001) EndoS, a novel secreted protein from *Streptococcus pyogenes* with endoglycosidase activity on human IgG. EMBO J. **20**, 3046–3055

Received 23 January 2013/10 July 2013; accepted 19 July 2013
Published as BJ Immediate Publication 19 July 2013, doi:10.1042/BJ20130126

© 2013 The Author(s)
The author(s) has paid for this article to be freely available under the terms of the Creative Commons Attribution Licence (CC-BY) (http://creativecommons.org/licenses/by/3.0/) which permits unrestricted use, distribution and reproduction in any medium, provided the original work is properly cited.
